# Overexpression of the KdpF Membrane Peptide in *Mycobacterium bovis* BCG Results in Reduced Intramacrophage Growth and Altered Cording Morphology

**DOI:** 10.1371/journal.pone.0060379

**Published:** 2013-04-05

**Authors:** Laila Gannoun-Zaki, Laeticia Alibaud, Séverine Carrère-Kremer, Laurent Kremer, Anne-Béatrice Blanc-Potard

**Affiliations:** 1 Laboratoire de Dynamique des Interactions Membranaires Normales et Pathologiques, Universités de Montpellier 2 et 1, CNRS-UMR5235, Montpellier, France; 2 INSERM, DIMNP, CNRS-UMR5235, Montpellier, France; French National Centre for Scientific Research - Université de Toulouse, France

## Abstract

Membrane peptides appear as an emerging class of regulatory molecules in bacteria, which can interact with membrane proteins, such as sensor kinases. To date, regulatory membrane peptides have been completely overlooked in mycobacteria. The 30 amino-acid-long KdpF peptide, which is co-transcribed with *kdpABC* genes and regulated by the KdpDE two-component system, is supposed to stabilize the KdpABC potassium transporter complex but may also exhibit unsuspected regulatory function(s) towards the KdpD sensor kinase. Herein, we showed by quantitative RT-PCR that the *Mycobacterium bovis* BCG *kdpAB* and *kdpDE* genes clusters are differentially induced in potassium-deprived broth medium or within infected macrophages. We have overexpressed the *kdpF* gene in *M. bovis* BCG to investigate its possible regulatory role and effect on mycobacterial virulence. Our results indicate that KdpF does not play a critical regulatory role on *kdp* genes expression despite the fact that KdpF interacts with the KdpD sensor kinase in a bacterial two-hybrid assay. However, overexpression of *kdpF* results in a significant reduction of *M. bovis* BCG growth in both murine and human primary macrophages, and is associated with a strong alteration of colonial morphology and impaired cording formation. To identify novel KdpF interactants, a mycobacterial library was screened using KdpF as bait in the bacterial two-hybrid system. This allowed us to identify members of the MmpL family of membrane proteins, known to participate in the biosynthesis/transport of various cell wall lipids, thus highlighting a possible link between KdpF and cell wall lipid metabolism. Taken together, these data suggest that KdpF overexpression reduces intramacrophage growth which may result from alteration of the mycobacterial cell wall.

## Introduction

Identification of short coding sequences is challenging, both experimentally and *in silico*, and functional natural peptides (defined herein as small proteins of size below 50 amino-acids) have been largely neglected in bacteria. However, recent studies conducted in Gram-negative bacteria have highlighted the regulatory role of small membrane proteins (for reviews see [1], [2]). These peptides seem to play a regulatory role by interacting with protein partners at the membrane, thereby modulating protein partner’s activity and/or stability. Some peptides, such as MgrR and SafA, have been shown to interact with the PhoQ sensor kinase [3], [4], [5], thus being able to modulate the expression of genes under the control of the corresponding two-component system. A *Salmonella typhimurium* peptide, MgtR, has been involved in the degradation of the MgtC virulence factor and thus contributes to *Salmonella* pathogenicity [6], [7]. There is often a lack of obvious phenotypes associated to the inactivation of small membrane ORFs but they can sometimes be unravelled following overexpression [4], [7], [8]. This suggests that such peptides might play a role for bacterial fitness under certain conditions or in specific environments, being otherwise involved in subtle regulatory mechanisms that are not essential to bacterial growth.

As for many other prokaryotes, except *E. coli* [9], [10], small proteins have been completely overlooked in mycobacteria so far and no global analysis has been carried out to predict or detect small mycobacterial ORFs. We have recently identified a 50 amino-acid-long membrane protein (Rv0900) that regulates expression of the OmpATb outer membrane protein at the post-transcriptional level [11]. To our knowledge, the only other known membrane peptide of *M. tuberculosis* is the 30 amino-acid-long KdpF peptide, which has been used to set up conditions for structural analysis of *M. tuberculosis* transmembrane domains [12]. The *kdpF* gene is the first gene of the *kdpFABC* operon. The Kdp system, which has been extensively studied in *E. coli*, is a P-type ATPase that transports K^+^ with high affinity [13], [14]. In *E. coli*, the KdpF peptide has been proposed to be a subunit involved in the stabilization of the KdpABC complex *in vitro*, possibly acting as a kind of lipid-like peptide for the holoenzyme [15]. However, the role of KdpF *in vivo* remains elusive because it is not essential for the function of the Kdp transporter [15]. Expression of the *kdpFABC* operon is dependent on external K^+^ concentration both in *E. coli* and *M. tuberculosis* [13], [14], [16], [17] and is regulated by the KdpDE two-component system, which is encoded by an adjacent operon. Interestingly, the KdpDE two-component system plays a role in *M. tuberculosis* virulence because a strain deleted for the *kdpDE* genes showed increased virulence [18]. Mouse infection studies have also shown a significant decrease in tissue colonization by an *M. paratuberculosis* mutant with a disruption in *kdpC* [19]. Overall, these studies underline a role of the *kdpABC* and *kdpDE* operons in the virulence of different pathogenic mycobacterial species. Moreover, the *kdpE* gene has been reported to be highly induced upon infection of macrophages [20] albeit these results have not been confirmed in a recent global expression analysis [21].

As a first step to investigate a putative regulatory role of the KdpF peptide towards the KdpDE two-component system, the *kdpF* gene was overexpressed in *M. bovis* BCG and the behaviour of this strain was subsequently studied in infected macrophages. Our results indicate that although KdpF does not act as an important regulator of the Kdp regulon, its overexpression in *M. bovis* BCG is accompanied by a significant decrease in the intracellular replication rate and by a pronounced alteration of bacterial cording.

## Materials and Methods

### Bacterial Strains and Growth Conditions

*Mycobacterium bovis* BCG Pasteur 1173P2 strain was grown on Middlebrook 7H10 agar (Difco) plates supplemented with Oleic Acid-Dextrose-Catalase (OADC) enrichment or in Sauton’s medium containing 0.025% tyloxapol (Sigma), in the presence of kanamycin (25 µg/ml) when required. Plates were incubated at 37°C for 2–3 weeks prior to visual counting of the colony forming units (CFU). Low potassium medium was obtained by replacing the potassium phosphate in the Sauton’s medium (2.87 mM) by a similar concentration of sodium phosphate. Strain used for cloning was *E. cloni* 10 G (Lucigen Corporation, Euromedex) that was grown in LB medium supplemented with kanamycin (25 µg/ml) at 37°C.

### Construction of a *M. bovis* BCG Strain Overepressing KdpF

The *kdpF* gene was PCR-amplified from H37Rv chromosomal DNA using the primers kdpF-Mtb-F-Msc and kdpF-Mtb-R-Eco (Table S1) and cloned into the pMV261 vector [22] at the *Msc*I and *Eco*RI sites. The resulting recombinant plasmid, p*kdpF*, was checked by sequencing and introduced, along with the empty pMV261 vector, by electroporation into wild-type *M. bovis* BCG. Recombinant strains were selected on Middlebrook 7H10 containing 25 µg/ml kanamycin after 3 weeks of incubation at 37°C and subsequently grown in liquid medium.

### Isolation and Infection of Human and Murine Primary Macrophages

Human blood samples, purchased from the local Blood Center (Montpellier, France), were collected from fully anonymized non-tuberculous control donors in accordance with French legislation. Purified monocytes, isolated as described [23] were seeded onto 24-well plates at a density of 7.10^5^/ml in complete culture medium (RPMI containing 10% FCS) and differentiated into macrophages with rh-M-CSF (10 ng/ml) (purchased from Al-Immuno tools) for 7 days. Human monocyte-derived macrophages (HMDM) were infected with exponentially growing *M. bovis* BCG cultures (DO_600_ = 0.8) at a multiplicity of infection (MOI) of 1∶1, as previously described [24].

Bone marrow-derived macrophages (BMDM) from 6 weeks-old C57BL/6 mice were prepared as previously described [25]. Animal experimentation was conducted in strict accordance with good animal practice as defined by the French animal welfare bodies and european ethic directive 2010/63/UE at the Institutional Animal Care of Montpellier (University Montpellier 2 and CNRS) in agreement with the Use Committee of the University Montpellier 2. BMDM were plated in 24-well plates at a density of 2.10^5^/ml and infected with exponentially growing *M. bovis* BCG cultures (DO_600_ = 0.8) at an MOI of 2∶1, as previously reported [24].

For bacterial RNA extraction from infected BMDM, 2.10^6^ macrophages were seeded into a 100 cm^2^ tissue culture dish and infected at an MOI of 10∶1. After a three-hour incubation period, cells were washed three times with phosphate buffer saline (PBS) medium and then incubated in DMEM medium. At day 6, cells were harvested, washed with PBS, lysed with PBS containing 0.1% Triton X100 and passed five times through a 26-gauge needle. Bacteria were then pelleted by centrifugation at 13 000 rpm for 10 min at 15°C. Total RNA was then isolated as described below.

### RNA Extraction and Quantitative RT-PCR (qRT-PCR)

RNA was prepared from 5 ml of mid-logarithmic bacterial cultures (grown in Sauton’s medium or grown for two days in Sauton’s medium deprived in potassium) or from bacteria recovered from infected-BMDM. Bacteria were harvested, resuspended in 1 ml of RNA protect reagent (Qiagen) and incubated 1 hr at room temperature. Bacteria were centrifuged and resuspended in 1 ml of RLT buffer from RNA easy kit (Qiagen), transferred in a Lysing matrix B tube (MP Bio) and disrupted with a bead-beater apparatus (3 times, 45 sec, maximal speed). RNA was purified with the RNA easy kit according to manufacturer’s instructions. DNA was further removed using DNAseI (Invitrogen). RNA integrity was analysed on a bioAnalyser 2100 (Agilent). cDNA was produced using Superscript III reverse transcriptase (Invitrogen). Controls without reverse transcriptase were done on each RNA sample to rule out possible DNA contamination. Quantitative real-time PCR was performed using an in-house SYBR Green mix [26] and a 480 light cycler instrument (Roche). PCR conditions were as follows: 3 min denaturation at 98°C, 45 cycles of 98°C for 5 sec, 68°C for 10 sec and 72°C for 10 sec. The *sigA* gene (*rv2703*) was used as internal control. The sequences of primers used for qRT-PCR are listed in Table S1.

### Bacterial Two-hybrid Analysis of KdpF-KdpD Interaction

The Bacterial Adenylate Cyclase Two-Hybrid (BACTH) system [27] was used to evaluate the interaction between KdpF and KdpD. The *kdpF* gene was PCR amplified using H37Rv chromosomal DNA as template and primers KdpF-Mtb-25Bam-F and KdpF-Mtb-25Eco-R (Table S1). The PCR fragment was cloned at the *Bam*HI and *Eco*RI sites of pKT25, to produce a fusion protein, KdpF-T25, where the T25 fragment is fused at the N-terminus of KdpF. The *kdpF* gene was also cloned in pKNT25 plasmid to produce a fusion protein where the T25 fragment is fused at the C-terminus of KdpF. To generate the pKT18-*kdpD* construct, the *kdpD* gene was PCR amplified using primers KdpD-Mtb-18Hind-F and KdpD-Mtb-18Bam-R using *M. tuberculosis* genomic DNA. The amplicon was then restricted by *Hind*III-*Bam*H1 and ligated into the pKT18 vector to produce a fusion protein, KdpD-T18. All constructs were verified by DNA sequencing.

Recombinant plasmids derived from pUT18 and pKT25 genes were co-transformed into BTH101 bacteria. Transformants were plated on LB broth with ampicillin and kanamycin (100 µg/ml, 50 µg/ml respectively) containing 50 µg/ml 5-bromo-4-chloro-3-indolyl-β-D-galactopyranoside (X-gal) at 30°C for 30 hrs. To quantify the interaction between hybrid proteins, bacteria were grown overnight at 30°C in LB broth containing 100 µg/ml ampicillin and 25 µg/ml kanamycin supplemented with 0.5 mM IPTG. The assays were carried out as described previously [28] with activities expressed in arbitrary Miller units. Values are average from at least four independent cultures. A level of β-galactosidase activity at least five-fold higher than the one of the control vectors indicates an interaction between the protein partners [29].

### Bacterial Two-hybrid Screen of KdpF Interactants

BTH101 competent cells harboring the *kdpF*-pKT25 bait plasmid were co-transformed with 1 µg of *M. tuberculosis* H37Rv DNA library in pUT18-vector (a generous gift from F. Bigi) as described [30]. Bacteria (2.4×10^5^) were plated at 30°C on M63 agar containing 0.3% lactose, ampicillin, kanamycin (50 µg/ml and 25 µg/ml, respectively) and 40 µg/ml of X-Gal. Blue colonies appearing after 5 days of incubation were tested at 37°C to increase the stringency and colonies that remained blue at 37°C were assumed to contain pUT18 clones coding for potential proteins interacting with KdpF. The pUT18 derivatives were then isolated and re-introduced into BTH101 bacteria containing *kdpF*-pKT25 to confirm the interaction. Plasmids from positives clones were further sequenced and analyzed with BLAST in the database.

The *mmpL7* gene as well as portions of the *mmpL7* gene were cloned in pUT18 vector using primers listed in Table S1. The portions of *mmpL7* gene code for transmembrane domains 2 to 6 or 8 to 12, and the second cytoplasmic domain (D2). The gene part coding for transmembrane domains 8 to 12 was also cloned into pUT18c vector to test the construction in other orientation in the membrane.

### Colony Morphology and Cording

Approximately 50 colony-forming units (CFU) were plated on cord-reading agar containing 0.0025% Triton X100 [31], incubated at 37°C for 3 weeks and observed under a light microscope MVX10 (Olympus). Single colonies were punched off the agar plate with the underlying substrate and immersed in 2.5% glutaraldehyde in 0,1 M sodium phosphate buffer pH 7.4 (PBS) and fixed for 2 hours at room temperature (RT); they were then washed in PBS and post fixed with 1% osmium tetroxide in PBS for 2 hours at RT. The fixed colonies were then washed in distilled water, dehydrated in a graded series of ethanol-water dilutions up to 100% ethanol, then exchanged for hexamethyldisilazane and dried on aluminum stubs. Samples were coated with 90Å of platinum in a Baltech SCD050 device. Scanning electron microscopy was performed on a FEI Quanta 200 FEG operated at 15 kV.

### Subcellular Distribution of PDIM in *M. bovis* BCG

Metabolic labeling of PDIM was performed by adding 1 µCi/ml of [1,2^−14^C] acetate (56 mCi/mmol, Amersham Biosciences) on *M. bovis* BCG cultures grown in Sauton’s liquid medium for 16 hrs at 37°C. Extraction of PDIM from subcellular fractions (culture supernatant, surface-exposed materiel, cytoplasmic and cell envelope) was performed as previously reported [32].

### Statistical Analysis

Statistical analyses were performed by EcoStats (http://ecostats34.free.fr), using R 2.14.0 (R Development Core Team, 2011). To account for potential variations between infection experiments, differences in count data of CFU per well between strains were analyzed using a generalized mixed effects model with a Poisson distribution, specifying « strain » as fixed factor and « experiment » as random factor. To test for differences in CFU all throughout the BMDM infection, « day » nested into « experiment » was used as random factor. Differences between ratio of *kdpA*, *kdpB*, *kdpD* and *kdpE* transcripts between strains were tested with generalized mixed effects models fitted with a beta distribution, specifying « strain » as a fixed factor and « gene » as random factor.

## Results and Discussion

### Construction of a *M. bovis* BCG Strain Overexpressing *kdpF*

**Figure 1:**
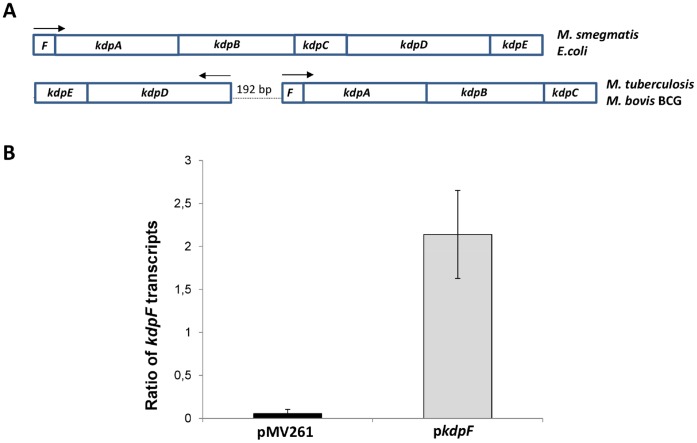
Genetic organization of the *kdp* operons in various bacteria and overexpression of the *kdpF* gene in *M. bovis* **BCG.** **A.** Transcriptional organization of the *kdp* operon in *E. coli* and various mycobacteria. An intergenic region of 192 bp separates the *kdpDE* operon from the *kdpFABC* operon in *M. tuberculosis* and *M. bovis* BCG. Arrows indicate the direction of transcription. **B.** Expression of *kdpF* transcripts relatively to those of s*igA* gene was measured by qRT-PCR from *M. bovis* BCG strains grown in Sauton’s medium carrying the pMV261 vector or p*kdpF* plasmid. Results are expressed as means ± SD from three independent experiments (each performed in triplicate).

The *kdpF* gene is the first gene of the *kdpFABC* operon, in both *E. coli* and *M. tuberculosis* (Fig. 1A). The KdpF peptides of *E. coli* and *M. tuberculosis* share only 30% identity (52% similarity), which is lower than the conservation found for the structural KdpA, KdpB and KdpC proteins (48 to 67% identity and 62 to 78% similarity). In *M. tuberculosis* and *M. bovis* BCG, the *kdpFABC* operon is adjacent to the *kdpDE* operon, but transcribed in the opposite direction, which contrasts with the gene organization in *E. coli* and *M. smegmatis* (Fig. 1A). The sequence of the *kdpF* gene, as well as the 192-bp regulatory intergenic region is identical between *M. tuberculosis* and *M. bovis* BCG. To investigate the regulatory role of KdpF and its putative effect on virulence, we have overexpressed the *kdpF* gene in *M. bovis* BCG. The *kdpF* gene was cloned in the pMV261 multicopy plasmid under the control of the *hsp60* promoter and the resulting plasmid, p*kdpF*, was introducted into *M. bovis* BCG Pasteur. Overexpression of *kdpF* gene results in a 40-fold increase in the level of *kdpF* transcript as shown by qRT-PCR (Fig. 1B).

### KdpF Interacts with KdpD but *kdpF* Overexpression Fails to Modulate the Regulation of *kdp* Genes by Potassium

**Figure 2:**
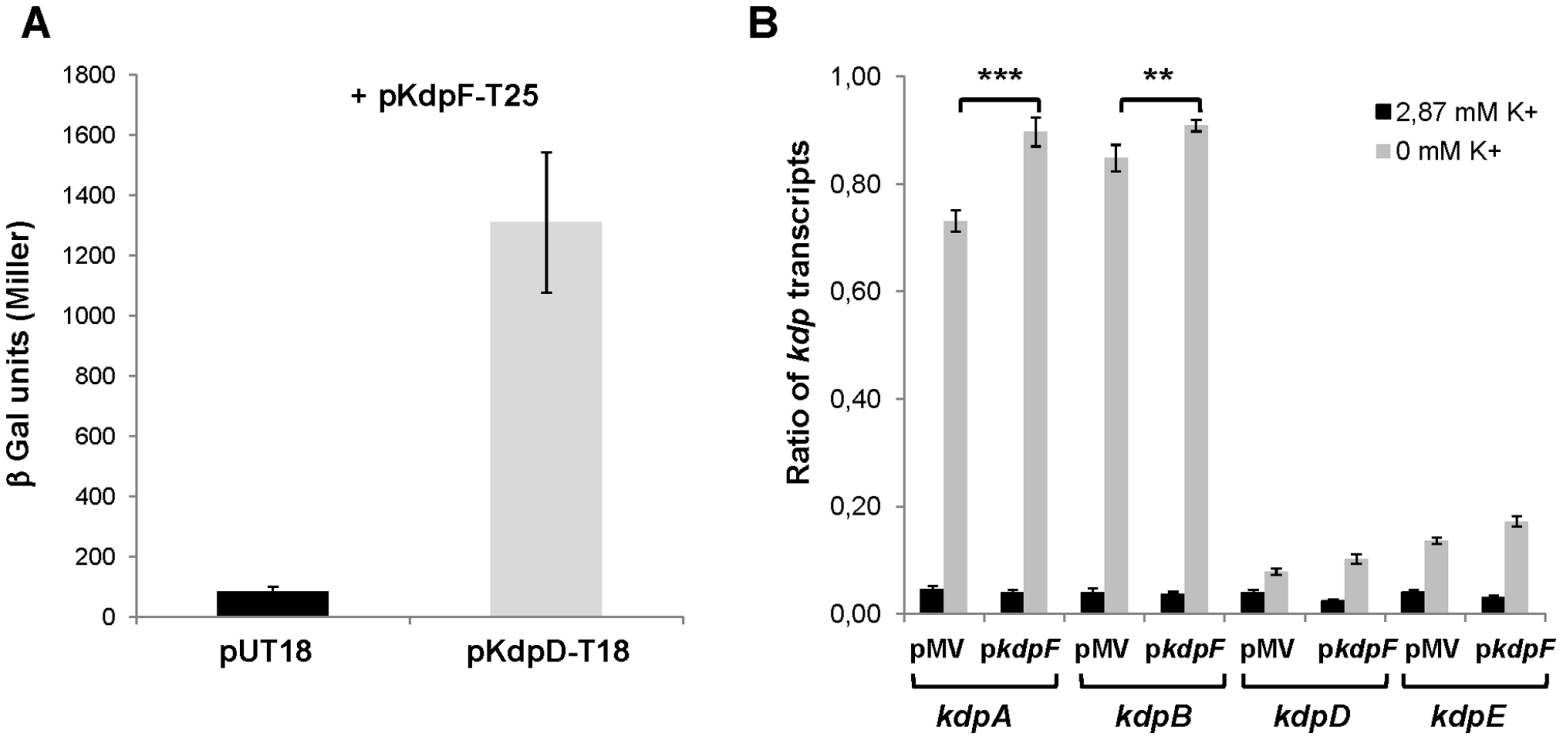
*In vivo* interaction of KdpF with KdpD and effect of *kdpF* overexpression on the regulation of *kdp* genes by K^+^. **A**. The interaction between KdpF and KdpD was assayed using the BACTH system by transforming *E. coli* BTH101 cells with KdpF-T25 and KdpD-T18 plasmids. Liquid β-galactosidase assays were performed from three independent experiments. Error bars represent standard deviations. **B.** K^+^-regulated expression of *kdp* operon. *M. bovis* BCG carrying the pMV261 vector or the p*kdpF* constructs were grown in Sauton’s medium or in K^+^-depleted Sauton’s medium. The levels of *kdpA, kdpB, kdpD* and *kdpE* transcripts relative to those of the *sigA* gene were measured by qRT-PCR. Results are expressed as means ± SD from three independent experiments (each performed in triplicate). Asterisks indicate statistical significance using a generalized mixed effects model (** P<0.01, *** P<0.001).

Recent studies have identified membrane peptides, MgrB and SafA, that interact with the PhoQ sensor kinase and modulate the regulation by this two-component system [3], [5]. To test whether the KdpF peptide could interact with the KdpD sensor kinase of the KdpDE two-component system, we have used the bacterial two-hybrid (BACTH) system that has been previously validated for detecting interactions between inner-membrane proteins in living bacteria [29]. Constructs were made to fuse the T18 or T25 fragment of adenylate cyclase to KdpD and KdpF, respectively. Both N- and C-terminal extremities of KdpD are cytoplasmic [16] (Fig. S1) and the T18 fragment has been fused at the C-terminal of KdpD (pUT18 vector). On the other hand, the topological orientation of KdpF was unknown and we have cloned the T25 fragment either at the KdpF N-terminal end (KdpF-T25; pKT25 vector) or C-terminal end (KdpF-NT25; pKNT25 vector). Plasmids encoding the T18 and T25 fusion proteins were introduced in an *E. coli cya* mutant (BTH101) and functional complementation was determined by measuring β-galactosidase activity (Fig. 2A). A high level of β-galactosidase activity (1300 Miller units) was observed when BTH101 was co-transformed with KdpD-T18 and KdpF-T25 plasmids, indicating a robust interaction between both proteins, whereas a basal level of β-galactosidase activity was measured with the negative control (T18/KdpF-T25). Moreover, this result indicates that the N-terminal extremity of KdpF is cytoplasmic (Fig. S1). Accordingly, a basal level of β-galactosidase activity was observed when T25 is fused to the C-terminal of KdpF (KdpF-NT25; data not shown), confirming that the C-terminal extremity of KdpF is periplasmic (Fig. S1).

We have next investigated whether the interaction between KdpD and KdpF might modulate the K^+^-mediated regulation by the KdpDE two-component system in *M. bovis* BCG. The KdpDE system regulates the *kdpFABC* operon, whose expression has been shown to be increased in low K^+^ medium in *M. tuberculosis* using a reporter gene fused to the *kdpF* gene [16]. In other bacteria as *E. coli*, the *kdpDE* genes follow the *kdpFABC* genes and are also regulated by KdpDE and by K^+^ [17]. In *M. tuberculosis*, the *kdpFABC* and *kdpDE* are divergent (Fig. 1A) and the regulation of *kdpDE* by K^+^ has not been studied. To measure the effect of *kdpF* overexpression on the K^+^-mediated regulation of *kdp* genes, we have monitored by qRT-PCR the transcription of both *kdpAB* and *kdpDE* genes in bacteria grown in Sauton’s medium containing 2.87 mM K^+^ or in K^+^-deprived Sauton’s medium. As expected for genes organized in operons, the *kdpA* and *kdpB* genes displayed the same expression pattern, as well as the *kdpD* and *kdpE* genes (Fig. 2B). The *kdpA* and *kdpB* genes were strongly induced (about 20 fold) in low K^+^ medium. The *kdpD* and *kdpE* genes are also induced by low K^+^, but to a much lesser extend than the *kdpAB* genes (only 3–4 fold). When the *kdpF* gene is overexpressed, the induction rate of *kdp* genes in low K^+^ medium is similar to the one observed with the pMV261 control vector, even though a very slight increase in the expression of *kdpA* and *kdpB* genes is observed (Fig. 2B). In addition, *kdpF* overexpression had no effect on the expression of an unrelated control gene *ompATb* (data not shown). Taken together, these results indicate that despite the fact that KdpF interacts with the KdpD sensor in the BACTH system, its overexpression does not importantly affect the regulation of gene expression by the KdpDE two-component system and therefore, KdpF does not appear to act as a major modulator of the KdpD sensor kinase. We then explored other phenotypes that may be modulated by KdpF overexpression, and particularly intramacrophage survival.

### KdpF Overexpression Reduces *M. bovis* BCG Intramacrophage Survival but Fails to Modulate Expression of *kdp* Genes Inside Macrophages

**Figure 3:**
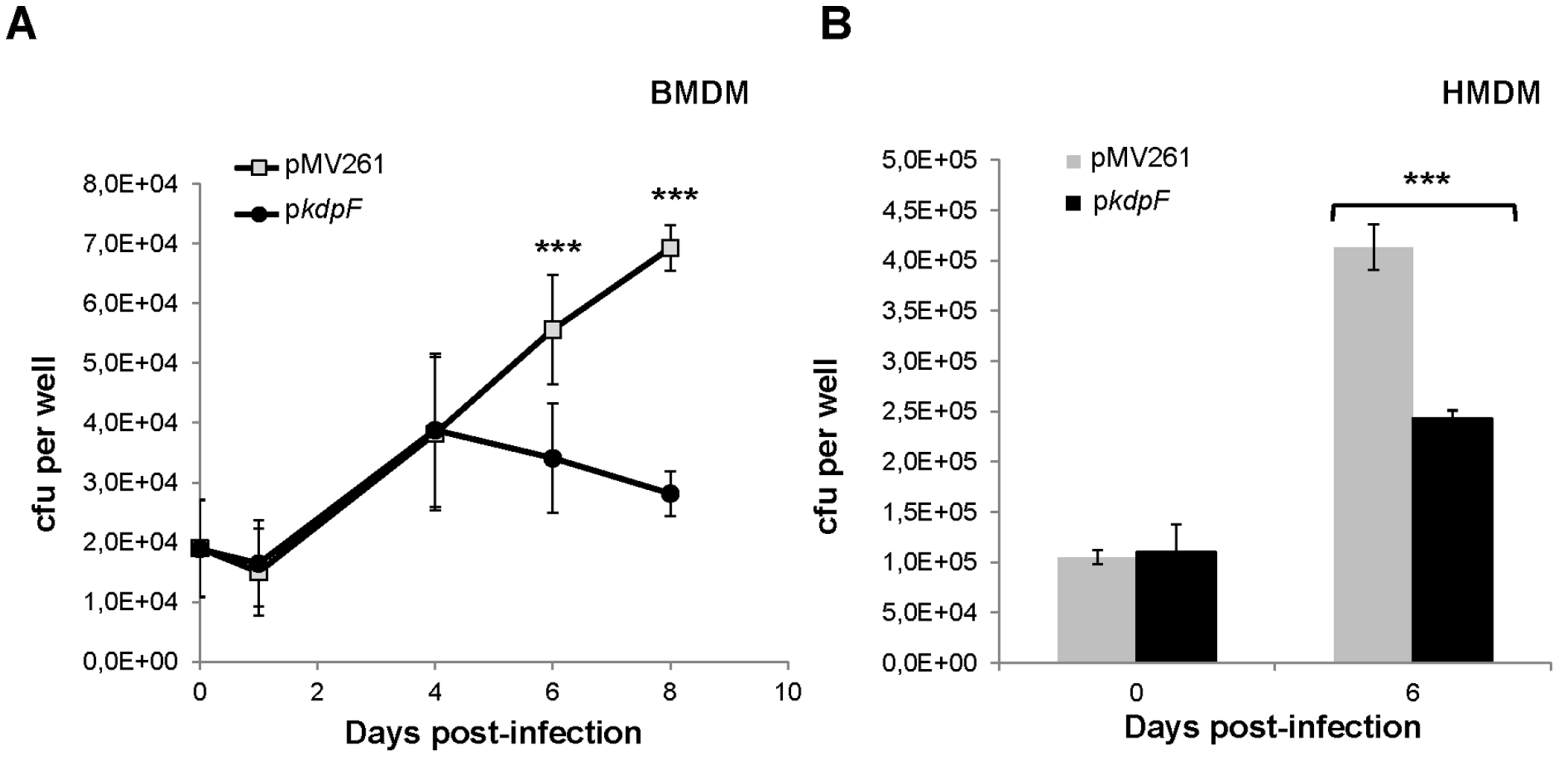
Growth of *M. bovis* BCG overexpressing *kdpF* in murine and human primary macrophages. **A**. Kinetic of growth of *M. bovis* BCG overexpressing *kdpF* in murine BMDM over a 8-day period. **B.** Bacterial number upon infection of HMDM infected with *M. bovis* BCG overexpressing *kdpF* at day 6 post-infection. The means ± SD calculated from three independent experiments (each performed in triplicate) are shown. Asterisks indicate statistical significance using a generalized mixed effects model (*** P<0.001).

We first investigated the survival of *M. bovis* BCG overexpressing or not *kdpF* in murine bone marrow-derived macrophages (BMDM) over an 8-day period. Results shown in Fig. 3A indicate that the bacterial multiplication is significantly lower in a strain overexpressing *kdpF*. The intracellular growth kinetic indicates that the defect does not occur at early stage of infection but appears 4 days post-infection, after a substantial replication of bacteria. A 40% decrease of bacterial multiplication was also observed upon infection of human monocyte-derived macrophages (HMDM) over a 6-day period (Fig. 3B), which is consistent with the profile obtained with BMDM. This growth defect of *kdpF*-overexpressing strain within macrophages is not due to an inherent growth defect of the strain because this strain grows similarly to the control strain carrying the pMV261 vector in Sauton’s medium (Fig. S2). In addition, both strains exhibited similar phenotypes regarding to tyloxapol or SDS susceptibility, as well as resistance to rifampicin, isoniazid or erythromycin (data not shown). This suggests that *kdpF* overexpression is very unlikely to alter cell wall permeability and that the lower growth in macrophages is not due to an early susceptibility to the bactericidal action of the phagosome.

**Figure 4:**
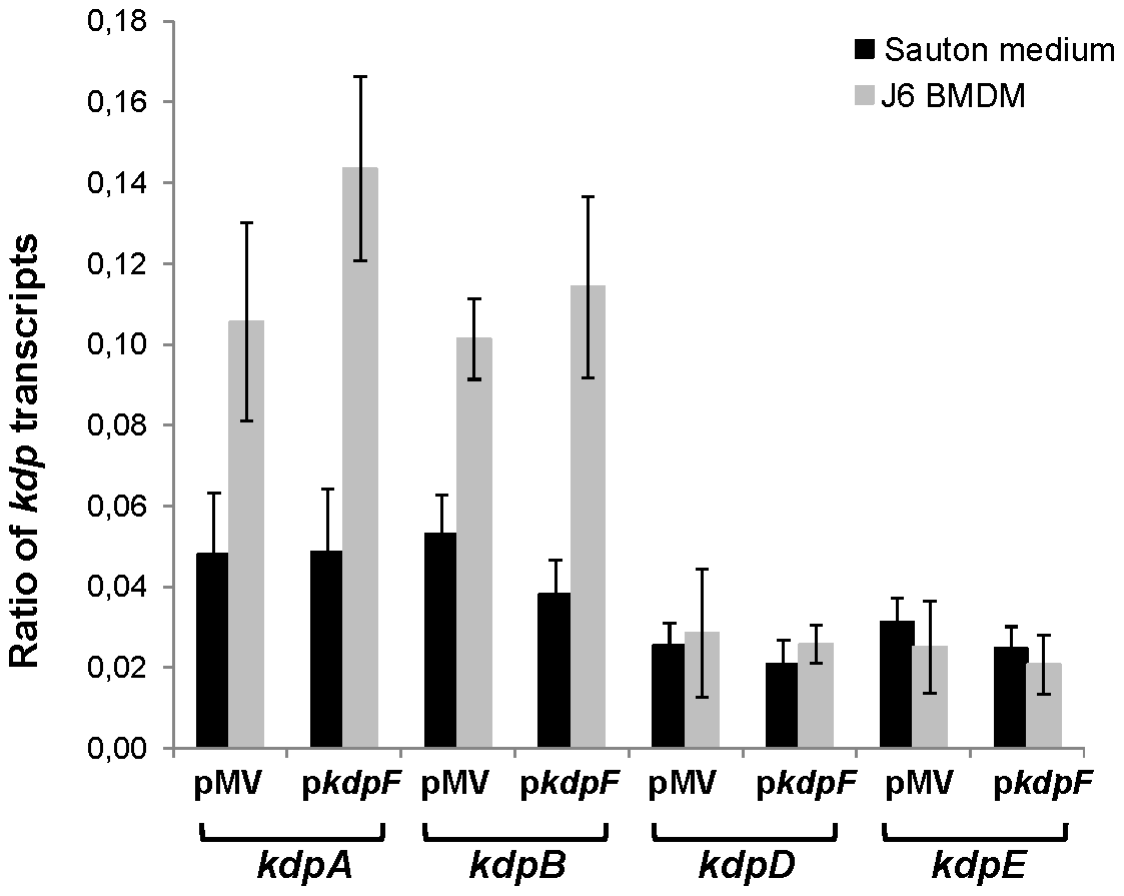
Effect of *kdpF* overexpression on the expression of the *kdp* operons inside macrophages. RNA was extracted from *M. bovis* BCG overexpressing *kdpF* grown Sauton’s liquid medium or after 6 days infection of BMDM. Quantitative RT-PCR was used to study the expression of the *kdp* operon genes relative to the one of the *sigA* gene. Data are means ± SD calculated from three independent biological samples analyzed in triplicate. Statistical significance was performed using a generalized mixed effects model.

Expression of the *kdp* regulon in macrophages was next investigated by qRT-PCR on bacterial RNA extracted six days post-infection. Our results indicate that *kdpA* and *kdpB* gene expression is mildly induced (about 2 fold) upon infection, as compared with expression in mycobacteria growing extracellularly in Sauton’s medium (Fig. 4). In contrast, *kdpD* and *kdpE* genes are not significantly induced in infected macrophages, consistent with the differential induction of *kdpAB* and *kdpDE* genes observed in low-potassium medium. These results suggest that the potassium concentration inside phagosome is not limiting, which is consistent with direct measurement of this cation [33]. That overexpression of *kdpF* does not alter the expression pattern of *kdp* genes, confirms that *kdpF* does not play a major regulatory role on the *kdp* regulon. This prompted us to search for proteins, unrelated to the *kdp* regulon, which may interact with KdpF.

### KdpF Interacts with MmpL7 in the Bacterial Two-hybrid System

**Figure 5:**
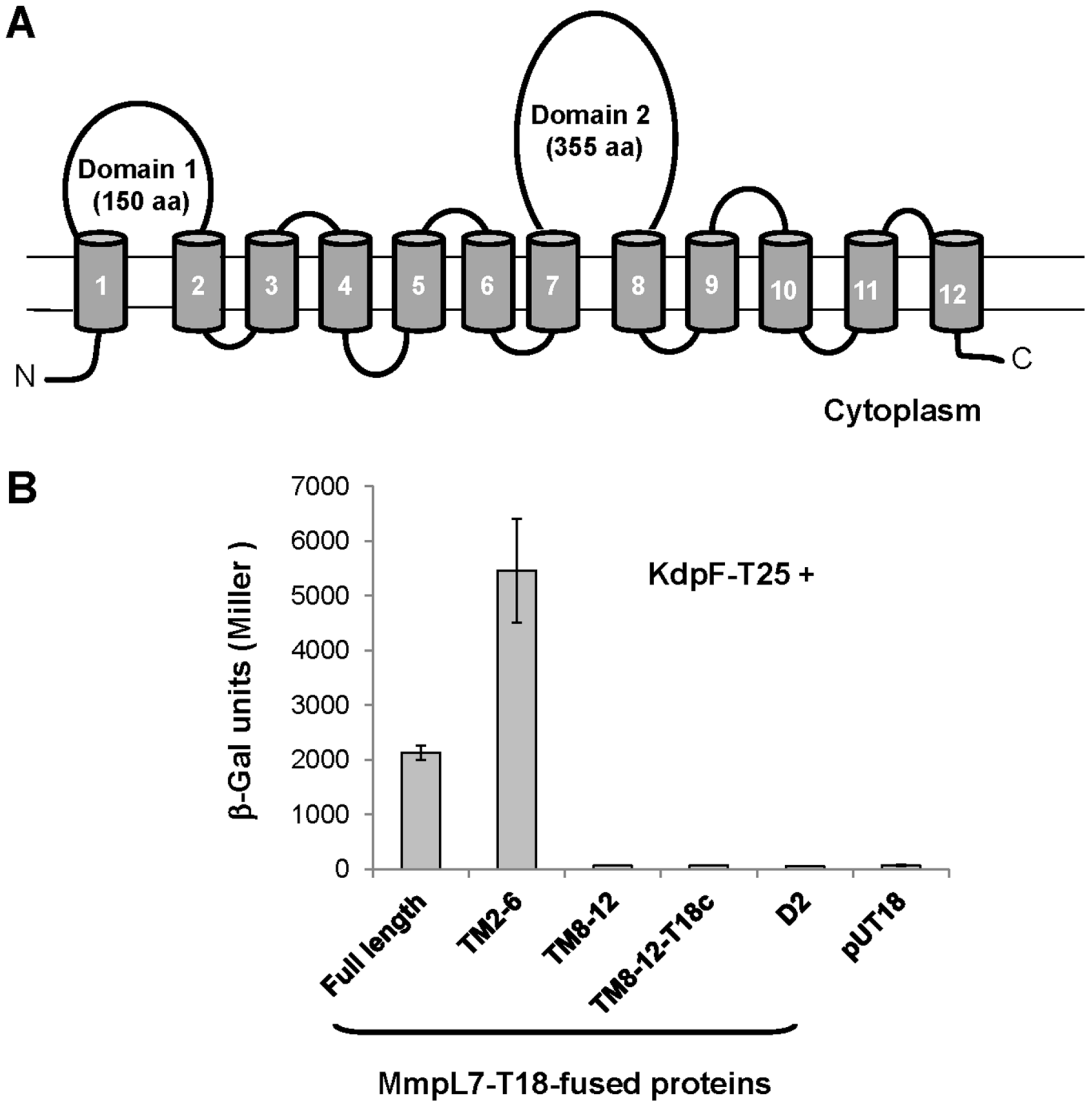
*In vivo* interaction of KdpF with MmpL7 variants using the BACTH system. **A**. Predicted topology of MmpL7 using the topological analysis server TMHMM (http://www.cbs.dtu.dk/services/TMHMM-2.0/). The TM regions are indicated by cylinders and are numbered while the two non-TM loops are indicated as Domain 1 and Domain 2. **B.** Interaction between KdpF-T25 and MmpL7-T18 constructs. *E.coli* BTH101 were co-transformed with *kdpF*-T25 plasmid and different constructs derived from pUT18 plasmid encoding MmpL7 full-length protein or portions of MmpL7 (TM 2–6, TM 8–12, cytoplasmic Domain 2). The portion coding for MmpL7 TM 8–12 was also cloned into pUT18c vector to obtain a reverse membrane orientation. The bars represent β-galactosidase activity expressed in Miller units ± SD of three independent experiments performed in triplicate.

To look for new KdpF interactants, a global genetic approach based on the BACTH system was performed by screening a *M. tuberculosis* DNA expression library cloned into pUT18 with KdpF used as bait (KdpF-T25). This screen led to the recovery of a portion of the MmpL10 transporter, corresponding to transmembrane (TM) domains 2 to 6 (amino-acids 220 to 387), in frame with the ORF encoding T18. Re-introduction of the clone into BTH101 bacteria containing *kdpF*-pKT25 generated homogenous blue colonies and β-galactosidase assays indicated high enzymatic activity (5780±826 Miller units, data not shown). However, for unknown reason, which may be linked to a dual orientation of the MmpL10 fragment, only a few blue colonies were recovered when the MmpL10 clone was co-transformed with *kdpF*-pKT25 in BTH101. MmpL proteins represent a family of 12 proteins [34], which share topological similarities including 12–13 transmembrane domains (Fig. 5A), involved in the biosynthesis and transport of complex lipids known to participate in various mycobacterial physiological functions [35], [36]. At least one of them, MmpL7, has been clearly involved in virulence [35], [37]. We have constructed several plasmids encoding the full length MmpL7 protein or portions of MmpL7 (TM 2–6, cytoplasmic domain 2, TM 8–12) fused to T18. The recombinant plasmids were co-transformed with KdpF-T25 plasmid in *E. coli* BTH101. The MmpL7-derived plasmid generated a high level of β-galactosidase activity (Fig. 5B). The interaction between KdpF and MmpL7 appears to be restrained to the transmembrane domains 2 to 6, since T18 fusions with the two other parts of the protein failed to interact with KdpF (Fig. 5B). Taken together, these results indicate that the KdpF peptide is likely to interact with the transmembrane domains 2 to 6 of MmpL7 and MmpL10.

### Analysis of Bacterial Morphology, Cording and Lipid Profiles upon KdpF Overexpression

**Figure 6:**
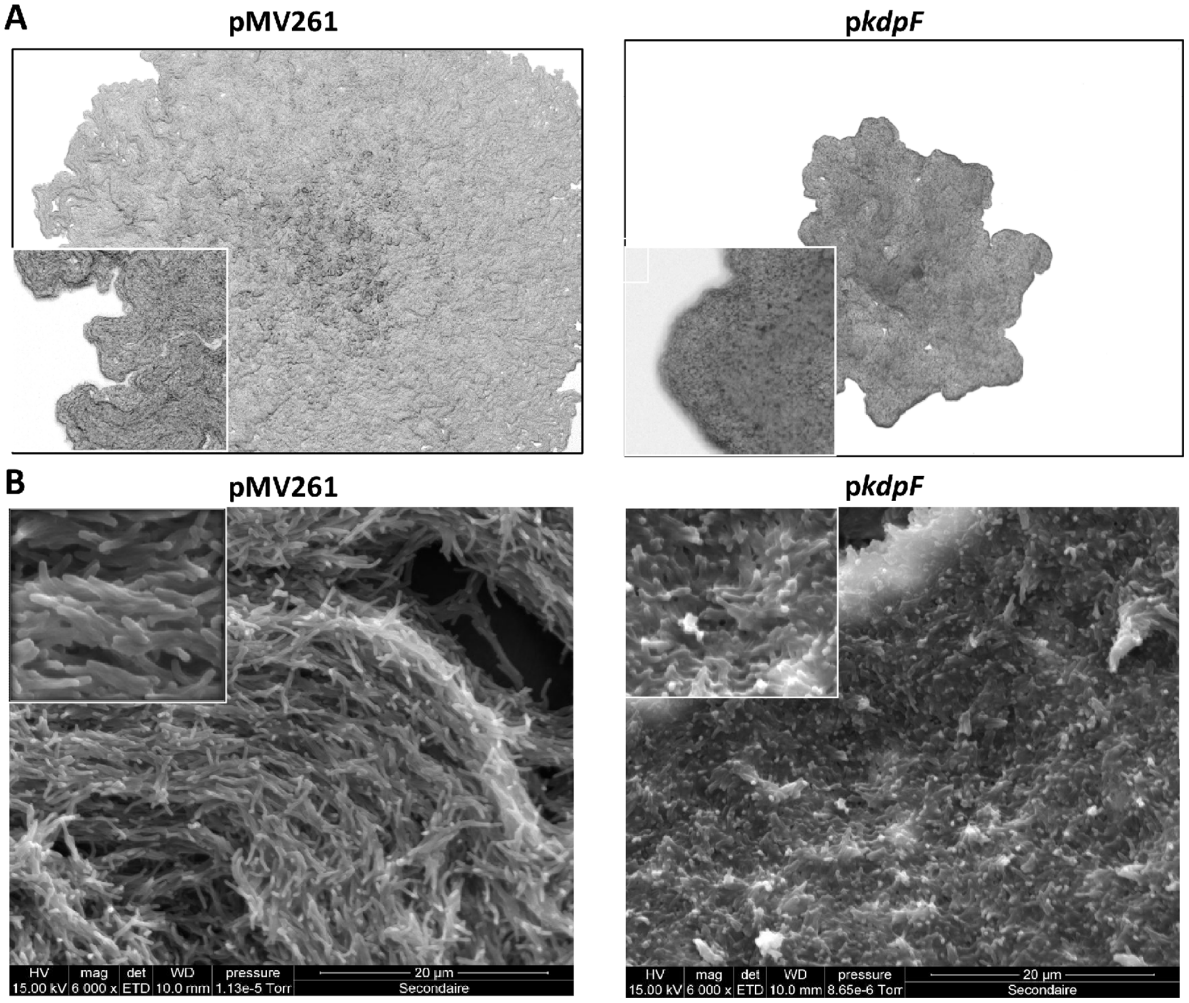
Morphotype of the KdpF-overexpressing strain. **A.** Single *M. bovis* BCG colonies were grown on cord-reading agar and visualized after 3 weeks. Magnification is 16x for the main figures and 63x for the insets. **B.** SEM micrographs of single *M. bovis* BCG colonies grown in 7H10 agar plates at 6000× magnification. The inset micrographs are at 13740x and 12000× magnification for *M. bovis* BCG pMV261 and *M. bovis* BCG overexpressing *kdpF,* respectively.

Despite the lack of growth phenotype of a *kdpF*-overexpressing strain in liquid medium (Fig. S2), we noticed that bacterial colonies were of smaller size on 7H10 solid medium (Fig. 6A). This effect was also associated with a different morphology which corresponds to a lower ability to form cords, as shown on triton X100 containing plates (Fig. 6A). Difference in the bacterial organization was clearly observed when the bacterial colonies from 7H10 plates were analyzed at high resolution (Fig. 6B). Scanning electron microscopy (SEM) of control strain (pMV261) revealed microscopic cords formed by organized bacteria. In contrast, the SEM micrographs of p*kdpF* colonies denoted an absence of microscopic cords and anarchical clustering of bacteria to form domes. Cording morphology, in which bacteria are intertwined into serpentine rope-like structures, is a feature of several mycobacterial species including *M. tuberculosis* [38] and *M. bovis* BCG [24]. In agreement with our data, a strong correlation has been found between microscopic cords and increased persistence of mycobacteria inside macrophages [24], [39].

Based on the intramacrophage growth defect and the morphological phenotype of a *kdpF*-overexpressing strain and owing to the specific interaction between KdpF and MmpL7/MmpL10, we hypothetized that *kdpF*-overexpressing strain may possibly present an altered cell wall lipid composition. MmpL7 has been involved in the transport of lipids, including the phthiocerol dimycoserosates (PDIM) [32], [37]. We, therefore, carried out cellular subfractioning and extraction of apolar lipids from strains overexpressing or not *kdpF* but failed to detect significant differences in the PDIM levels (Fig. S3). More generally, thin layer chromatography profile failed to detect differences in apolar lipids (data not shown). We cannot exclude the possibility of changes in the composition of (glycol)lipids that are not separated in the solvent system used or a defect occurring specifically when bacteria grow on solid medium or reside in macrophages. In addition, our bacterial two-hybrid screen suggests that KdpF can interact with several membrane proteins. Hence, the growth defect of *kdpF*-overexpressing strain in macrophages and alteration in bacterial cording could result from a larger network of interactions of KdpF with multiple cell wall proteins. Cumulatively, these results indicate that KdpF overexpression alters bacterial morphology and cording but the modulation, if any, in cell wall composition that underlies these modifications remains unknown.

### Concluding Remarks

Membrane peptides have been recently ascribed as a novel class of regulatory molecules in bacteria, which includes members that modulate the activity of two-component systems [1], [2]. In addition, some natural membrane peptides can exhibit anti-virulence properties when promoting the degradation of virulence factors [7]. The present results, obtained with *M. bovis* BCG, indicate that the KdpF peptide does not have an important regulatory role towards the KdpDE two-component system. However, our study denotes the ability of KdpF to restrict intramacrophage bacterial growth and ability to form bacterial cords when overexpressed. This may be linked to an alteration of the KdpABC transporter function and/or a modulation of the function of other membrane proteins, including lipid transporters, which could interact with KdpF. Hence, KdpF appears as an attractive membrane peptide since it displays anti-virulence functions upon overexpression in mycobacteria.

## Supporting Information

Figure S1 **Topology of KdpD and KdpF.** Both N- and C-terminal ends of KdpD are cytoplasmic and the T18 fragment has been fused to the C-terminal end. KdpF has a cytoplasmic N-terminal end since interaction with KdpD is observed only when the T25 fragment is fused to this extremity.
(TIF)

Figure S2 **Growth curve of** ***M. bovis*** **BCG overexpressing** ***kdpF*** **grown in Sauton’s liquid medium over a 15 days period.** Bacteria were diluted from exponentially growing cultures with an initial OD_600_ of 0.02. The graph is representative of four independent experiments.
(TIF)

Figure S3 **One-dimension autoradiographic TLC of [1,2-^14^C]acetate-labeled apolar lipids.** *M. bovis* BCG strains harboring pMV261 or p*kdpF* plasmid were grown in Sauton’s liquid medium and labeled with 1 µCi ml^−1^ of [1,2^−14^C] acetate and further incubated for 16 hrs at 37°C with gentle agitation. Cultures were fractionated and equal amount of radiolabeled lipids from each fraction were applied onto a TLC plate, developed using petroleum ether/acetone (49∶1, v/v) and exposed to a Kodak Biomax MR film for 7 days. Fractions are indicated as follows: culture supernatant (Sup), surface-exposed materiel (S1), cytoplasmic and plasma membrane (S2) and cell wall component (pellet). Purified PDIM A and PDIM B prepared from *M. marinum* were used as standards following charring with molybdophosphoric acid (not shown).
(TIF)

Table S1 **Oligonucleotides used in this study. F and R stand for forward and reverse, respectively.**
(DOC)
