# Supplementary figures and images for: Overexpression of the KdpF Membrane Peptide in Mycobacterium bovis BCG Results in Reduced Intramacrophage Growth and Altered Cording Morphology
Source: PLoS One. 2013 Apr 5;8(4):e60379. doi: 10.1371/journal.pone.0060379 (PMC3618439; doi:10.1371/journal.pone.0060379)

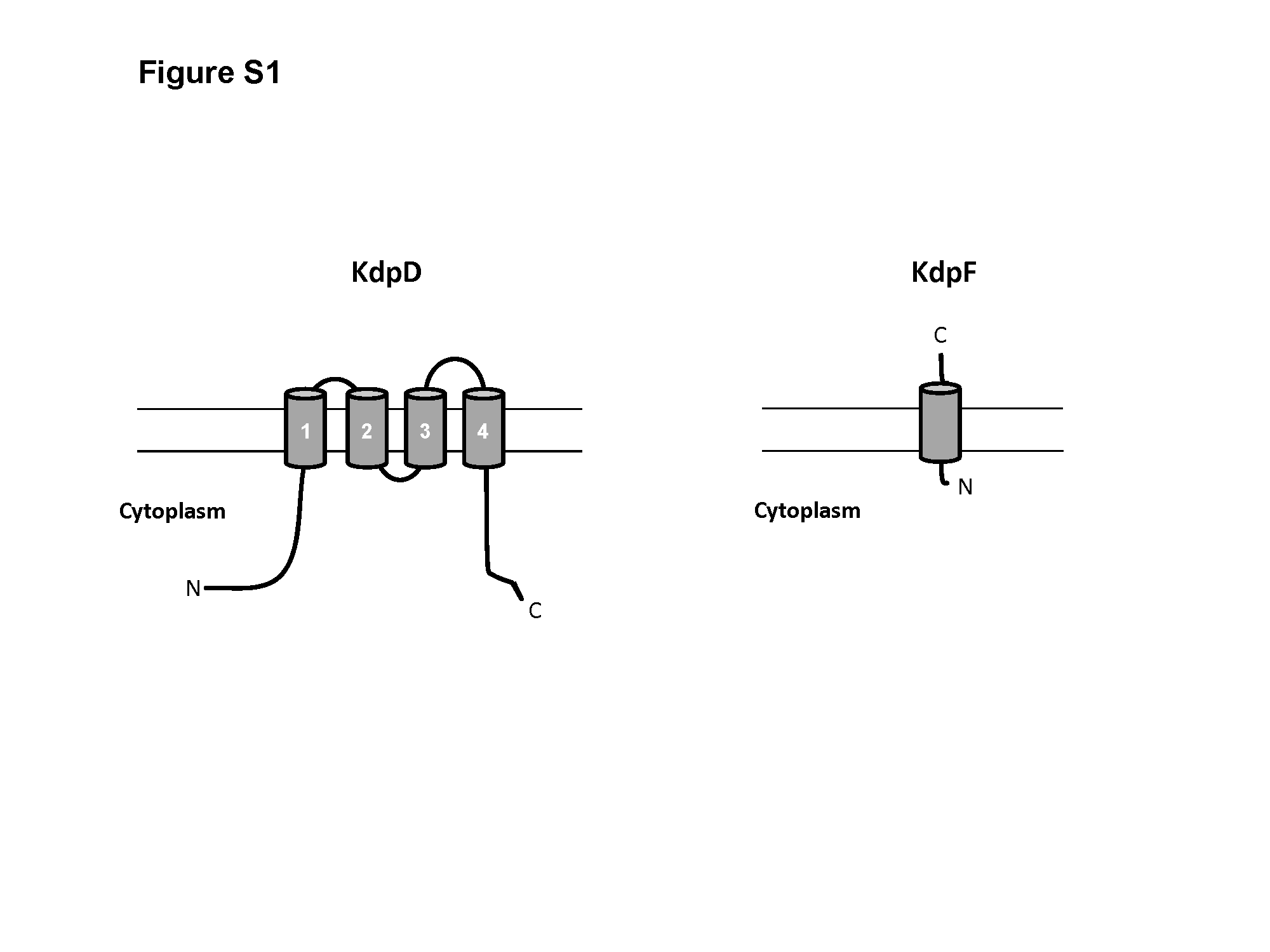

Supplement: Figure S1 — Topology of KdpD and KdpF. Both N- and C-terminal ends of KdpD are cytoplasmic and the T18 fragment has been fused to the C-terminal end. KdpF has a cytoplasmic N-terminal end since interaction with KdpD is observed only when the T25 fragment is fused to this extremity. (TIF) [file pone.0060379.s001.tif]

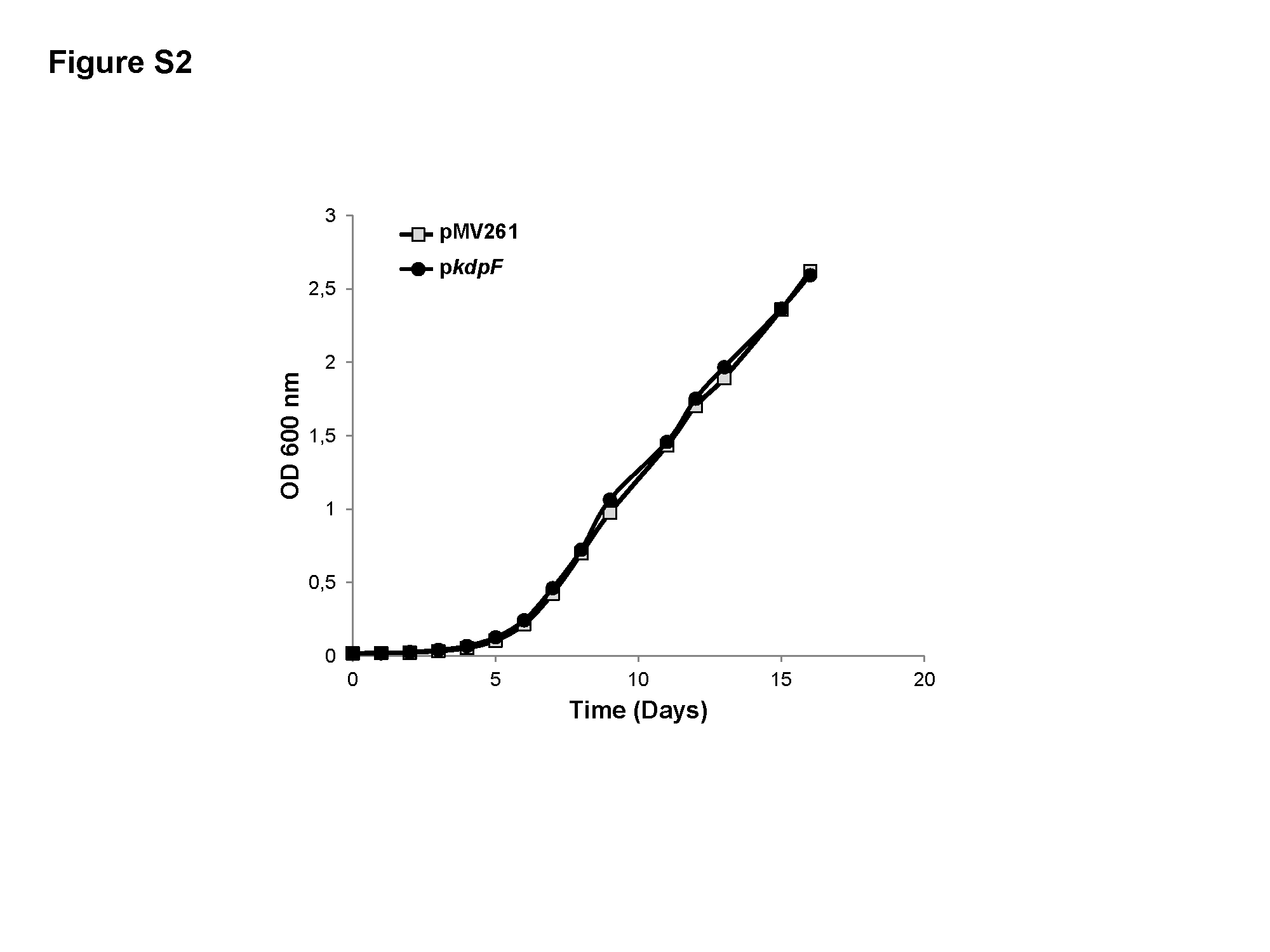

Supplement: Figure S2 — Growth curve of M. bovis BCG overexpressing kdpF grown in Sauton’s liquid medium over a 15 days period. Bacteria were diluted from exponentially growing cultures with an initial OD600 of 0.02. The graph is representative of four independent experiments. (TIF) [file pone.0060379.s002.tif]

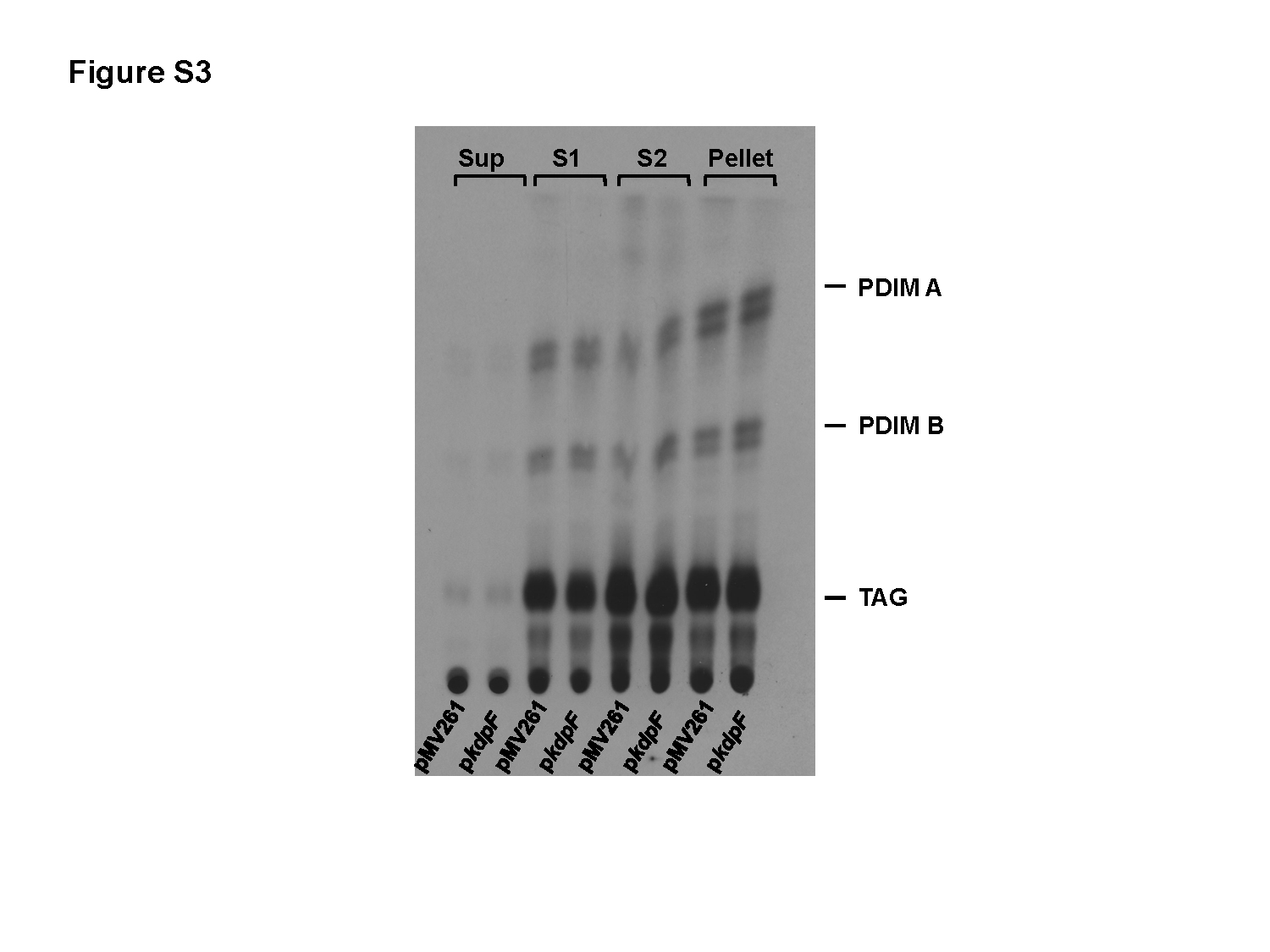

Supplement: Figure S3 — One-dimension autoradiographic TLC of [1,2-14C]acetate-labeled apolar lipids. M. bovis BCG strains harboring pMV261 or pkdpF plasmid were grown in Sauton’s liquid medium and labeled with 1 µCi ml−1 of [1,2−14C] acetate and further incubated for 16 hrs at 37°C with gentle agitation. Cultures were fractionated and equal amount of radiolabeled lipids from each fraction were applied onto a TLC plate, developed using petroleum ether/acetone (49∶1, v/v) and exposed to a Kodak Biomax MR film for 7 days. Fractions are indicated as follows: culture supernatant (Sup), surface-exposed materiel (S1), cytoplasmic and plasma membrane (S2) and cell wall component (pellet). Purified PDIM A and PDIM B prepared from M. marinum were used as standards following charring with molybdophosphoric acid (not shown). (TIF) [file pone.0060379.s003.tif]
