# Supplementary material for: Overexpression of the KdpF Membrane Peptide in Mycobacterium bovis BCG Results in Reduced Intramacrophage Growth and Altered Cording Morphology
Source: PLoS One. 2013 Apr 5;8(4):e60379. doi: 10.1371/journal.pone.0060379 (PMC3618439; doi:10.1371/journal.pone.0060379)
Supplement: Table S1 — Oligonucleotides used in this study. F and R stand for forward and reverse, respectively. (DOC) [file pone.0060379.s004.doc]

**Supporting Table**

**Table S1:** Oligonucleotides used in this study. F and R stand for forward and reverse, respectively.

| **Primer** | **Sequence (5’-3’)** |
| --- | --- |
| KdpF-Mtb-25Bam-F | CGGGATCCCatg actacggtcgacaacatcgtc |
| KdpF-Mtb-25Eco-R | GGAATTCTCAGAACTTCTCCGGAAACAGCAGC |
| KdpF-Mtb-NT25Eco-R | GGAATTCGAGAACTTCTCCGGAAACAGC |
| KdpD-Mtb-18Hind-F | CCCAAGCTTGgtgacgttgctcttcgccgatc |
| KdpD-Mtb-18Bam-R | CGGGATCCTCTGGGCGGTCCTCGGGAGCCGCC |
| kdpF-Mtb-F-Msc | CAATGGCC ATGACTACGGTCGAC |
| kdpF-Mtb-R-Eco | CCGGAATTCTCAGAACTTCTCCGG |
| mmpL7-Mtb-pUT18-Hind-F | CCCAAGCTTGatgcctagtccggctggcc |
| mmpL7-Mtb- pUT18-Xba-R | GG TCT AGA GTACGCCGCCCTGGCGTGGTC |
| MmpL7-TM2-6-Mtb-18-Hind-F | CCC AAG CTT G Acc gtt gcg gcg gtg atc gcc gtc |
| MmpL7-TM2-6-Mtb-18-Bam-R | CG GGA TCC TC AGA GAC GGG TAG CGA CAA CCG GCC TG |
| MmpL7-TM8-12-Mtb-18-Hind F | CCC AAG CTT G caa ata gcc gcg gct gtc cgc g |
| MmpL7-TM8-12-Mtb-18-Bam-R: | CG GGA TCC TC ACG CCG CCC TGG CGT GGT CGG |
| MmpL7-TM8-12-Mtb-18C-Pst-F | AA CTGCAG G caa ata gcc gcg gct gtc cgc g |
| MmpL7-TM8-12-Mtb-18C-Sal-F | AA GTC GAC G caa ata gcc gcg gct gtc cgc g |
| MmpL7-D2-Mtb-18-Hind-F | CCC AAG CTT G gcc gag aac ccg aca agg caa ggc |
| MmpL7-D2-Mtb-18-Bam-R | CG GGA TCC TC CGC GGC CCC ACC CAC CGT GAC CTG |
| SigA-RT-F | gcccgaggagctggccaaag |
| SigA-RT-R | ccaagctggctgtcgccctc |
| kdpF-RT-F | ACGGTCGACAACATCGTCGGG |
| kdpF-RT-R | ACAGCAGCGCCGCGAATAGG |
| kdpA-RT-F1 | CGCTGGGGGTGACACAGCAG |
| kdpA-RT-R1 | GACCGAGCCGTTGGAGGTGC |
| kdpB-RT-F1 | CGCTTCGACGAAATGCGCCG |
| kdpB-RT-R1 | GCAAGCTTGTCCTCGGGCGT |
| kdpD-RT-F2 | CGATATGCGCCCGACTGCGT |
| kdpD-RT-R2 | TCATGGGCGGTCCTCGGGAG |
| kdpE-RT-F1 | GGTATCGACGTGCTCGGCGG |
| kdpE-RT-R1 | CACCGGCTGCTCCAACTCGG |
| OmpA-RT-intF2 | ccgggtagccgacaagctc |
| OmpA-RT-R2 | ccacgatctcgacgcgacg |
